# 表观遗传修饰调控微小RNA表达在肺癌中的研究进展

**DOI:** 10.3779/j.issn.1009-3419.2020.102.29

**Published:** 2020-07-20

**Authors:** 锡麟 胡, 凯华 田

**Affiliations:** 266071 青岛，青岛大学附属医院胸外科 Department of Thoracic Surgery, The Affiliated Hospital of Qingdao University, Qingdao 266071, China

**Keywords:** 肺肿瘤, miRNA, 表观遗传修饰, Lung neoplasms, miRNA, Epigenetic modifications

## Abstract

肺癌是全世界癌症引起死亡中较常见的一种。近年来，参与肺癌发病的分子机制被逐步揭开，但是其发生发展的确切机制并未完全阐明。其中微小RNAs（microRNAs, miRNAs）是一种短小并且广泛存在于植物、病毒及人类等各种生物中的内源性单链的非编码RNA。miRNAs在正常肺组织中发挥着多种功能，它参与细胞生长、代谢、增殖和分化等众多生物学过程。而miRNAs的异常表达与肺肿瘤的发生、发展、侵袭、转移相关。因此，miRNAs可被视为一种新的生物标志物。与编码蛋白质的基因类似，miRNA的表达和功能受多种因素以及表观遗传网络（包括DNA甲基化和组蛋白修饰机制）的调控。此外，miRNAs本身也能调控那些表观遗传修饰的关键酶来影响表观修饰。miRNA与表观基因学之间的相互联系将有助于我们研发以miRNA为导向的肺癌诊断、治疗和预后的方案。

## Abstract (en)

Lung cancer is one of the primary causes of cancer-induced death among the world. Although the network of molecular implicated in lung cancer is gradually revealed, the exact molecular mechanism of its occurrence and development has not been fully elucidated. As a class of small and endogenous single-stranded non-coding RNAs, microRNAs (miRNAs) are found in a wide range of organisms from plants, viruses to humans. miRNAs involve various functions in normal lung tissue development. They take part in a large amount of biological processes including cell growth, metabolism, proliferation and differentiation. However, aberrant expression of miRNAs could induce the occurrence, development, invasion and metastasis of lung tumor, so it is deemed the novel biomarkers. Similar to that of protein-coding genes, expression and function of miRNA are regulated by various factors and the epigenetic network which includes DNA methylation and histone modification. Moreover, key enzymes driving epigenetic modifications are regulated by miRNAs. Therefore, better understanding of inextricable linkage between miRNAs and epigenome will provides a basis for the feasibility of miRNA-orientated diagnostic, therapeutic and prognostic strategies related to lung cancer in future.

## 1 前言

肺癌是全世界癌症引起死亡中最常见的一种，5年生存率仅为18%。其中80%的肺癌患者由于诊断较晚而无法进行手术。在美国，每年大约有22万人被诊断为肺癌和支气管癌，主要诱因是吸烟^[1]^。近年来，第二代测序技术的进步与发展使我们在理解肺癌发展的分子机制方面取得了巨大进展。表观遗传模式可以在不改变DNA序列的前提下改变可见表型^[2]^，同时参与对基因表达的调控和维持基因组的稳定性^[3, 4]^，因此在肺癌的发展中起着关键作用。这些机制不仅调控蛋白质编码基因，而且影响蛋白质非编码基因^[2]^。微小RNAs（microRNAs, miRNAs）是由发夹结构产生的小的非编码RNA。生物信息学技术的发展与成熟促使着miRNA研究的进展，其在表观遗传调控、肺癌细胞生长周期的调控、细胞分化与凋亡等方面的作用正在被揭示^[5-7]^。表观遗传学调控着miRNA表达，而miRNA的异常表达可以诱导肺癌的发生、发展、侵袭、转移。这促使了人们寻找和评估这些分子，以用于癌症的诊断和治疗。本综述将对表观遗传学机制和miRNA之间的关系和在肺癌中的作用进行综述。

## 2 表观遗传学与肺癌

遗传和表观遗传改变的结合，引起基因表达和功能的失调，可能是癌症发生的重要原因之一^[8]^。因此，表观遗传模式的改变以及遗传异常可以使我们对肿瘤的发生有一个更好的了解。癌症中最著名的表观遗传调控是启动子区域CpG岛的异常甲基化以及组蛋白修饰中的去乙酰化/甲基化改变^[8]^。

DNA甲基化通常是甲基与CpG岛的胞嘧啶残基和5-甲基胞嘧啶结合^[9]^。约60%的人类基因启动子都能观察到这些修饰。甲基的加成反应是由DNA甲基转移酶（DNA methyltransferases, DNMTs）催化的。已知的DNMTs有3类，根据它们的底物类型和产生的功能来区分：DNMT1、DNMT3A和DNMT3B。在正常细胞中，甲基化是纠正基因表达模式、细胞分化和发育的重要途径之一^[10]^。然而，在肿瘤组织中，因为癌基因或抑癌基因相关的关键调控区受到表观遗传修饰，DNA甲基化的稳态可能被破坏。已经发现，肿瘤在DNA甲基化改变上表现出比正常组织更高的特异性，包括低甲基化和高甲基化^[11]^。低甲基化与基因组的不稳定性和印迹丢失、非整倍体以及癌基因的激活有关^[12]^。然而，DNA高甲基化可能因为靶基因在人类基因组中的位置的不同而发挥不同作用。在基因启动子内的CpG丰富区，高甲基化是导致多种肿瘤中大量的抑癌基因沉默的原因^[13]^。DNA高甲基化诱导转录沉默主要有两种机制：①甲基化的CpG岛阻碍了转录因子与DNA序列的结合，或阻碍了抑制蛋白如组蛋白脱乙酰酶（histone deacetylases, HDACs）的形成。DNA高甲基化可能引起甲基化的DNA与具有高亲和力的蛋白质复合物结合成甲基结合域复合物，进而直接或间接地阻碍转录因子的结合，影响基因表达^[14]^；②甲基CpG结合蛋白，如mbd和mep2可以诱导抑制基因表达的作用^[15]^。而在肺癌细胞中，某些抑癌基因CpGs区域的甲基化以及正常基因组内广泛低甲基化，可致抑癌基因沉默和染色体旋转程度的增加，从而引起肿瘤细胞的生长^[16, 17]^。据报道，吸烟可能会对DNA甲基化产生影响，甚至在戒烟之后的几年内仍然有效^[18]^。观察肺癌患者全血DNA样本发现疾病相关基因存在异常的甲基化^[19, 20]^，芳香烃受体阻遏子基因和表达凝血酶或胰蛋白酶受体3的*F2R*基因的低甲基化与与吸烟和肺癌风险增加有关^[19]^。同时，有研究^[18]^发现肺癌患者支气管上皮细胞的p16和死亡相关蛋白激酶的启动子甲基化程度较低。在吸烟者和未患肺癌的前吸烟者的支气管上皮细胞中也存在这种异常甲基化，因此这种低甲基化可能与吸烟相关。

另一种基因表达受表观调控的方法是组蛋白修饰，即通过改变组蛋白尾，来实现基因表达的动态调节^[21]^。最常见的组蛋白修饰有乙酰化、甲基化、泛素化、磷酸化和蔗糖化^[21]^。组蛋白修饰调节基因表达主要有两种方式：①组蛋白乙酰化影响转录活性；②组蛋白甲基化导致染色质浓缩与失活^[22]^。组蛋白的乙酰基团在HDAC作用下能够被消除，进而诱导基因沉默。HDAC在细胞分化、增殖和凋亡中发挥着关键作用^[23]^。同时这种消除可能与肺癌的病程进展相关，因为许多癌症分子具有改变HDAC的活性的作用^[24]^。

目前以表观遗传学为作用靶点的药物主要包括HDAC抑制剂、DNMT抑制剂和Janus激酶2（Janus kinase 2, JAK2）抑制剂。DNA甲基化被认为是肺癌强有力的治疗靶点，其中在实验和临床上最广泛使用的药物是DNMT抑制剂，如阿扎胞苷和地西他滨^[25-27]^。

## 3 miRNA与肺癌

引起癌症的表观遗传学重要因素还有非编码RNA，这些RNA不被翻译成蛋白质，而在转录水平和转录后水平上调节基因的表达^[28, 29]^，并被认为是调控癌症等疾病发生的关键因子，因此许多非编码RNA被列为致癌因素或肿瘤抑制因子^[30]^。miRNAs是一种长度为19 nt-24 nt的单链非编码RNA。它们是在1993年对秀丽杆线虫的研究中发现的，随后迅速地被认识到，高度保守的miRNA序列在单细胞和多细胞真核生物之间的调节途径中起着至关重要的作用^[31]^。miRNAs具有调控转录后基因表达，促进mRNAs切割或者抑制mRNAs翻译的作用^[32, 33]^。正常生理下，miRNA的生物合成受到机体严格的控制，但是癌症能够解除这种控制^[34]^。解除对miRNA表达的控制会导致其功能的改变。过表达的miRNAs和低表达的miRNAs可能分别作为促进肿瘤发展的癌基因和抑制肿瘤发展的抑癌基因。此外，由于调控目标和生物功能的多样性，在一种肿瘤亚型中作为癌基因的miRNA可能在另一种肿瘤亚型中起着抑制癌细胞的作用^[35]^。

基因缺失、表观遗传修饰、广泛的转录抑制或生物合成功能障碍是肿瘤中成熟miRNA表达异常的主要原因。miRNA分子的表观遗传修饰，主要包括胞嘧啶或腺嘌呤甲基化，可影响RNA的稳定性、定位、剪接和靶向性^[36]^。比如，在29%的肺癌患者中发现CpG岛的甲基化可以影响对miR-34a的调控^[37]^。在Belinsky等^[38]^关于小细胞肺癌的研究中，HAS-miR-137和HSA-miR-886-3P的调节也受到启动子甲基化的影响。

有研究^[39]^表明装载miRNA-126的外泌体（231-Exo）通过中断PTEN/PI3K/AKT信号通路明显抑制A549肺癌细胞的增殖和迁移。此外，静脉内施用载有miRNA-126的外泌体可在小鼠中产生有效的肺归巢效应。Zhang等^[40]^发现环状RNA circFGFR1通过与癌细胞中的miR-381-3p相互作用来促进非小细胞肺癌进展和对抗基于程序性细胞死亡1（programmed death-1, PD-1）的治疗。Wang等^[41]^发现N6-甲基腺苷诱导的miR-143-3p/VASH1轴在肺癌的脑转移中起着关键作用。具体数据见表 1。因此，miRNA与肺癌细胞的发生发展、转移、治疗有着密切的关系。

**1:** miRNA表达在肺癌中的应用 Application of miRNA expression in lung cancer

Study	miRNA	Mechanism	Application
Zhang PF^[40]^	miR-381-3p	circFGFR1	Prognostic value
Wang H^[41]^	miR-143-3p	VASH1	Development of metastasis
Wei D^[50]^	miR-30a-3p	p38-MAPK	Treatment
Cui H^[54]^, Sun Y^[55]^, Liu B^[56]^	miR-126	EGFL7，VEGF	Dignosis
Gallardo E^[57]^	miR-34a	CDK 4/6, CCND1, MYCN and Sirtuin1	Prognostic value
Tan W^[58]^	miR-127	Bcl-6	Prognostic value
Kim NH^[65]^	miR-34b/c	p53	EMT
Chen Y^[66]^	miR-148a	DNMT1	Development of metastasis
Watanabe K^[63]^	miRNA-34b	EGFL7	Development of metastasis
Oshima G^[71]^	14q32 miRNA	DNMT1, DNMT3B	Development of metastasis
Garzon R^[72]^, Fabbri M^[73]^	miR-29	DNMT3A/3B, DNMT1	Treatment
Yu F^[75]^	miR-124a	USP14	Treatment
EMT: epithelial-to-mesenchymal transition; USP14：ubiquitin-specific protease 14; miRNA: microRNA.			

## 4 表观遗传学与miRNA

miRNA主要通过作为表观遗传机制的一部分，以蛋白质编码基因相同的机制进行自我的表观遗传学修饰。表观遗传修饰主要包括组蛋白乙酰化和甲基化以及miRNA基因启动子甲基化。Wang等^[42]^发现大约50%的miRNA基因与CpG岛相关，这意味着它们的表达可以被DNA甲基化所调控。在人类癌症中，CpG岛的启动子高甲基化是抑癌基因miRNA沉默的最常见原因之一^[43]^。miRNA在癌症中特定的表达揭示着肿瘤特定的甲基化模式^[44]^。同时，miRNA的表观遗传学调控也揭示出表观遗传效应物和miRNA之间的特异性^[45]^。除了甲基化，用于结合甲基的CpG结合域蛋白也被发现可以调控miRNA的转录^[44]^。因此，肿瘤细胞中DNA的甲基化影响miRNA的表达，可用于确定肿瘤分类、临床诊断、预后及治疗反应。此外，组蛋白修饰也能影响miRNA的表达谱，来使抑癌基因沉默^[46]^。比如，组蛋白的甲基化和HDAC过表达，它们可以激活或抑制miRNA的表达^[47]^。这表明miRNA的表达和功能受多种因素以及表观遗传网络（包括DNA甲基化和组蛋白修饰机制）的调控。此外，miRNA本身也能调节表观遗传修饰，从而在细胞生物学中发挥关键作用^[48]^。比如，epi-miRNAs能够调控包括DNMTs和HDACs在内的重要表观基因调节因子的表达，同时建立一种精确的调控反馈机制^[47, 49]^。Wei等^[50]^发现miR-30家族的miR-30a-3p通过对A549细胞中的DNMT3A的作用来调节p38-MAPK通路，从而抑制肺癌的进展，这暗示着miR-30a-3p可能是治疗肺癌的一种新的潜在治疗策略。

## 5 miRNA：肺癌的诊断

肺癌的早期诊断对于改善患者预后极为重要，能大大降低患者的死亡发病（81%）^[51]^。肺癌患者血清或组织中的miRNA的特定表达，结合胸部CT可为早期肺癌的诊断提供帮助。Keller和同事^[52]^发现miR-126是用于区分非小细胞肺癌组与健康对照组较合适的miRNA。在一项涉及86例非小细胞肺癌和57例健康对照组的研究中，miR-126在肿瘤患者血浆中表达水平降低了59.09%-68.18%。同时血浆中的miR126将非小细胞肺癌与健康对照人群区分开来的敏感性和特异性分别为73%和96%^[53]^。Cui等^[54]^发现在多种癌症中存在miR-126基因启动子的甲基化增强，从而引起miR-126表达的降低。低表达的miR-126导致靶向调节EGFL7来抑制非小细胞肺癌细胞增殖的作用和下调VEGF来抑制肺癌细胞生长的作用减弱^[55, 56]^。因此，miR-126的异常表达与非小细胞肺癌的发病有关。

## 6 miRNA：评估肺癌的预后

表观遗传学影响miRNA表达可作为评估癌症复发风险的新方法，同时也是有助于指导治疗决策的有用工具。miR-34家族由miR-34a、miR-34b和miR-34c组成。它们是p53网络的一部分，在DNA损伤或致癌因素作用下，p53可诱导miR-34家族的表达。此外，在大量癌症患者中，miR-34a的表达与*p53*突变和miR-34a基因启动子区域的甲基化相关。miR-34a的表达与复发和总生存相关。低水平的miR-34a表达预示着复发的可能性较高。多变量分析也表明miR-34a是复发的独立预后指标^[57]^。此外，miR-127能够以原癌基因*bcl-6*为作用靶点，bcl-6的主要作用是调节DNA损伤引起的凋亡反应。当miR-127因表观遗传改变沉默时，bcl-6将被激活^[58, 59]^。Tan等^[58]^研究发现miR-127的表达改变在肿瘤中的变异是肿瘤存活和生长的重要介质，肿瘤细胞中miR-127甲基化修饰与肿瘤的存活与分期都有独立的联系，预示着不良的预后。Liu等^[60]^利用TCGA中的公共RNA序列、DNA甲基化和miRNA序列数据，建立了肺腺癌样品中的DNA甲基化-miRNA-基因相互作用网络发现95个与预后相关的表观遗传相互作用中，25个相互作用在高甲基化水平/高miRNA表达水平（H/H）和低甲基化水平/低miRNA表达水平（L/L）之间表现出显著差异。此外，肿瘤组织中的表观遗传生物标记物miR-1290、miR-196b和miR-135a的组合以及血浆中的miR-21、miR-25、miR27b和miR-326可用来预测晚期非小细胞肺癌患者铂类化疗的疗效^[61]^。

## 7 miRNA：肺癌的侵袭和转移

肿瘤细胞转移主要是指肿瘤细胞脱离原发生长部位，在机体内远离原发部位的器官或组织中继续增殖生长，形成同样性质的肿瘤。主要途径有淋巴道转移、血道转移和种植性转移。miRNA与肺癌转移的相关机制：（1）miRNA与上皮间质转化（epithelial mesenchymal transitions, EMT）相关。上皮间质转化是指上皮细胞失去上皮表型，转化为间充质细胞，同时获得间质细胞表型的一种生物学现象。在肺癌表观遗传学改变中，miR-34a和miR-34b/c的表达被DNA甲基化所抑制^[57, 62, 63]^，41%的原发性非小细胞肺癌和67%的原发性小细胞肺癌患者的miR-34b/c被甲基化^[63, 64]^。在肺癌中，miR-34/p53轴通过影响Snail家族基因的表达来调控EMT^[65]^。Chen等^[66]^发现miR-148的DNA甲基化与肺癌转移有关。非小细胞肺癌细胞miR-148b表达低于正常支气管上皮细胞。miR-148的作用主要有：①可引起上皮相关钙黏蛋白E表达增加，间质相关钙黏蛋白N和波形蛋白表达减少；②调节下游TGF-信号转导因子ROCK1，抑制细胞增殖和EMT^[67]^。因此，miR-148b的甲基化能促进EMT。（2）miRNA可以调节肺癌细胞信号转导途径，进而影响肿瘤转移。Watanabe等^[63]^检测了99例原发性非小细胞肺癌的DNA甲基化状态，发现miRNA甲基化与组织学侵袭性和淋巴浸润有显著相关。其中H1975和Calu-1细胞系中的miRNA-34b被其宿主基因*EGFL7*的自身启动子的DNA甲基化所沉默。在肺组织中，miRNA-34b/c在p53抑癌途径中起重要调节作用。因此，miRNA-34b的DNA甲基化可以作为肺癌侵袭性表型的生物标志物。miRNA-148a是肺癌组织中一种常见的失调miRNA。Chen等^[66]^对非小细胞肺癌miR-148a表观遗传调控的研究发现miR-148a编码区的高甲基化导致非小细胞肺癌中miR-148a的表达水平降低，而miRNA-148a能够抑制DNMT1的活性。由于DNMT1对维持细胞DNA甲基化模式至关重要，这将导致甲基化敏感基因失活，包括转移抑制蛋白（例如钙黏蛋白E）^[68, 69]^。在多种侵袭性癌症中发现钙粘蛋白E的功能丧失与转移和侵袭相关^[70]^，因此，miR-148a与非小细胞肺癌的淋巴结转移、晚期临床分期、总生存期缩短密切有关。此外，分解DNA甲基转移酶DNMT1和DNMT3B以及使用地西他滨来抑制DNA甲基化能够调节对癌转移有抑制性的14q32 miRNA的表达。在肺癌和肝转移的临床前模型中，甲基化依赖性14q32 miRNA的表达抑制了转移定植，并与转移性癌症患者的临床结局改善相关。这些发现暗示了DNA甲基化的表观遗传修饰通过miRNA网络调节癌转移，并揭示了地西他滨对转移抑制性miRNA激活的作用^[71]^。miRNA表达的表观遗传学改变可以用于研究抑制肺癌转移的潜在治疗靶点，从而提高肺癌患者的生存率。

## 8 miRNA：肺癌的治疗

对于具有癌基因或抑癌基因特点的miRNA，可通过外源性地消除或导入，来降低或提高其细胞内水平，进而达到抑制肺癌细胞生长的作用。①miRNA可与传统抗癌药物联合使用。miR-29家族直接以DNMT3A/3B为作用靶点或通过Sp1以DNMT1为作用靶点，可通过Src-ID1通路在肺癌中起抗增殖和抗转移的作用^[72, 73]^。因此，在肺癌细胞株中，miR-29s表达具有恢复表观遗传修饰的正常模式和重新激活甲基化沉默的抑癌因子的作用^[73]^。Sacko等^[74]^还将染料木黄酮与miRNA-29b共包裹在混合纳米粒中，因两种分子能够攻击多个靶点，这种联合治疗模式具有优异的抗增殖作用；②miRNA调控可用于辅助靶向治疗。已有报道说明肺癌中存在miR-124基因的甲基化^[43]^。miR-124a能通过调控靶基因泛素特异性蛋白酶14（ubiquitin-specific protease 14, USP14）的表达抑制肺癌干细胞的生长和自我更新，并促进其凋亡和提高吉非替尼敏感性。因此，外源的miR-124a和USP14可用于辅助非小细胞肺癌的靶向治疗^[75]^。Wei等^[50]^发现miR-30家族的miR-30a-3p通过对A549细胞中DNMT3A起作用来调节p38-MAPK通路，从而抑制肺癌的进展，这表明miR-30a-3p可能是针对肺癌的一种新的潜在治疗策略。

## 9 结论

随着对miRNA在肺癌发生发展中的作用和分子机制研究的不断深入，miRNA可以用于识别高危人群，区分良性肿块和恶性肿瘤及治疗。同时，miRNA不仅能够在转录后水平上起调控作用，而且也是表观遗传机制中的调控对象。这个复杂网络的各成分之间的异常失衡与肺癌的发生机制、转移浸润及治疗方案有重要联系。因此，更好地理解miRNA和表观遗传学的相互作用，将提高我们对肺癌发生过程的认识，在不久的将来帮助我们找到可以用来征服肺癌的有效策略。
